# Treatment-modifying effects of frailty on stroke reperfusion therapy outcomes: a systematic review and meta-analysis

**DOI:** 10.1093/ageing/afag080

**Published:** 2026-04-10

**Authors:** Anakha Varma, Shiv Bhakta, Esmee Dohle, Elizabeth Warburton, Nicholas R Evans

**Affiliations:** Department of Clinical Neurosciences, University of Cambridge, Cambridge, England, United Kingdom of Great Britain and Northern Ireland; Department of Clinical Neurosciences, University of Cambridge, Cambridge, England, United Kingdom of Great Britain and Northern Ireland; Department of Clinical Neurosciences, University of Cambridge, Cambridge, England, United Kingdom of Great Britain and Northern Ireland; Department of Clinical Neurosciences, University of Cambridge, Cambridge, England, United Kingdom of Great Britain and Northern Ireland; Department of Clinical Neurosciences, University of Cambridge, Cambridge, England, United Kingdom of Great Britain and Northern Ireland

**Keywords:** acute ischaemic stroke, frailty, thrombolysis, thrombectomy, outcomes, older people, systematic review

## Abstract

**Background:**

Frailty is common amongst individuals presenting with acute ischaemic stroke (AIS). Not only has frailty been found to have disease-modifying effects in terms of survival and disability after AIS, but it may also exert a treatment-modifying effect in reperfusion therapies. However, studies investigating this to date have frequently been of limited sample size, highlighting the potential for meta-analysis to definitively establish any treatment-modifying effect.

**Objectives:**

We investigate the effect of pre-stroke frailty on morbidity and mortality outcomes following reperfusion treatment (thrombectomy and thrombolysis) for AIS.

**Methods:**

A systematic review was performed according to Preferred Reporting of Items in Systematic Reviews and Meta-Analyses guidelines, via searching the EMBASE, PubMed, Scopus and Web of Science databases up to August 2025.

**Results:**

We identified 11 relevant studies with 194,699 participants. Overall, the prevalence of frailty was 37.2% [frail (n = 72,311), non-frail (122,096)]. Frailty was associated with increased 90-day (RR 2.19 [95% CI 1.44–3.34]) and one-year mortality (RR 2.11 [95% CI 1.6–2.78]), but not with symptomatic intracranial haemorrhage (RR 1.23 [95% CI 0.78–1.96]) or modified Rankin score 3–5 (RR 2.20 [0.94–5.16]).

**Conclusions:**

Frailty has a consistent association with mortality at different time points after AIS reperfusion therapies. Despite some study heterogeneity, there is evidence that pre-stroke frailty is associated with increased mortality after treatment, though not with increased risks of symptomatic intracerebral haemorrhage or post-stroke disability. These findings suggest that routine pre-morbid frailty assessment may inform the decision-making process for AIS reperfusion treatment administration. This study highlights the need for large multi-centre prospective trials.

## Key Points

- Pre-stroke frailty assessment is highly variable in terms of both indexing method and threshold for frailty definition.
- There is a significant increase in 90-day and one-year mortality outcomes post-reperfusion therapies for acute ischaemic stroke in patients with pre-morbid frailty.
- However, pre-morbid frailty was not found to be associated with an increased risk of complications relating to reperfusion therapies (post-treatment symptomatic intracranial haemorrhage, pneumonia).
- Standardised assessment of pre-stroke frailty may inform prognostication and clinical decision-making in the hyper-acute setting.

## Introduction

Frailty is a clinical syndrome characterised by a decline of physiological capacity to respond to stressor events [1]. Although distinct entities, frailty commonly increases with age [2], and has been identified as an independent risk factor for increased morbidity and mortality across a range of medical conditions, including cardiovascular disease [3, 4]. Stroke represents an archetypal acute stressor event, with increasing evidence that pre-morbid frailty has an adverse effect on stroke severity and outcomes [5–7]. Crucially, frailty can be used as a proxy for physiological age, which does not necessarily correlate with its chronological counterpart, as evidenced by the independent association between frailty and mortality following stroke (both ischaemic and haemorrhagic), irrespective of the individual’s chronological age [8–10].

Current reperfusion therapies in acute ischaemic stroke (AIS) include intravenous thrombolysis and endovascular mechanical thrombectomy. Intravenous thrombolysis, using tissue plasminogen activator, is most effective when administered within 4.5 hours of stroke onset, and aims to dissolve the thrombus to restore blood flow. Endovascular thrombectomy involves the mechanical removal of a thrombus causing a large vessel occlusion. Both treatments have been shown to improve functional outcomes and reduce disability [11, 12]. However, despite the increasing recognition of the role of frailty in stroke, evidence for the impact of pre-stroke frailty on the efficacy of reperfusion therapies in AIS is sparse and largely confined to relatively small observational studies.

Establishing whether pre-stroke frailty has a treatment-modifying effect on reperfusion therapies is of vital importance. Frailty is present in one in four individuals presenting with stroke, but this prevalence may rise to around three-quarters when pre-frailty is considered [6, 13]. This proportion may rise over the coming decades due to the ageing population and predicted rise in the number of strokes [7, 14], with the consequent rise in concurrent frailty having important implications for the delivery of stroke services. However, recognition that carefully selected older individuals continued to benefit from reperfusion therapies shows the importance of avoiding treatment nihilism based simply on chronological age [15]. This has been reinforced by the shift in clinical and research practice towards the importance of assessing factors beyond chronological age, such as frailty, in order to optimise selection of individuals with AIS who are likely to benefit from reperfusion therapies [16, 17].

Although frailty has not been used to determine eligibility for research studies investigating reperfusion treatments, frequently the stringent functional inclusion criteria (based on good pre-morbid function as measured by the modified Rankin scale, mRS) has meant that many individuals with frailty have been excluded by proxy. Consequently, results from these studies are unlikely to be directly applicable to a large proportion of cases presenting in clinical practice.

Numerous frailty scoring systems exist as a means to evaluate frailty, such as the Clinical Frailty Score (CFS), Hospital Frailty Risk Score (HFRS) and Frailty Index. The use of different tools used to measure frailty in stroke cohorts may reflect the time available for assessment (such as time-dependent prospective assessment versus retrospective assessment) and the availability of relevant information for assessment [18]. The heterogeneity of tools used, and lack of consensus on which assessment to use, has hindered the implementation of a standardised assessment of frailty in the hyperacute stroke setting.

This systematic review aims to establish the range of frailty assessment tools used in studies investigating hyperacute stroke reperfusion therapies, the outcomes measured in these studies and to summarise the treatment-modifying effect of frailty on reperfusion therapies in AIS. Understanding the impact of pre-stroke frailty on the benefits and risks of treatment has the potential to inform prognostication and clinical decision-making for individuals with concurrent frailty and acute ischaemic stroke.

## Methods

This systematic review was reported in accordance with the Preferred Reporting of Items in Systematic Reviews and Meta-Analyses (PRISMA) guidance [19]. The protocol was registered in 2023 on the International Prospective Register of Systematic Reviews (PROSPERO CRD42023420145).

Each aspect of the screening review process was conducted by a minimum of two reviewers with third party referral as required.

### Search strategy and selection criteria:

We placed no restriction on date of publication, inclusive to August 2025. Further inclusion criteria refer to studies reported with:

(1) Adult populations having suffered an acute ischaemic stroke with reperfusion therapies (thrombolysis and/or thrombectomy).
(2) Defined frailty scoring tools.
(3) Human study participants.
(4) English language publication.

Identified abstracts were used to search for full-text publications using author names, and were subsequently assessed. If no full-text was available, then abstracts were excluded.

Further exclusion criteria include:

(1) Studies not reporting quantitative data on frailty prevalence in accordance with reperfusion therapies.
(2) Studies not reporting frailty scoring prior to stroke and therapeutic intervention.
(3) Studies reporting outcomes only after transient ischaemic attack (TIA), haemorrhagic stroke, subarachnoid or subdural haemorrhage.
(4) Non-primary study designs (e.g. reviews, meta-analyses, case report, chapters, opinion editorials).
(5) Full-text unavailable.

### Population

We included studies with adult participants (>16 years old) who presented with AIS and who underwent attempted reperfusion with thrombolysis and/or thrombectomy for acute ischaemic stroke.

### Exposure

Studies have investigated frailty using various scoring models, including (but not limited to) CFS, HFRS and electronic frailty index. We included any author-described frailty scoring system, provided the intention was to quantify frailty prior to stroke. The majority of studies analysed outcomes using dichotomised frailty measures, and for the purpose of our analysis, we used these dichotomised groups pre-specified by the authors. Where the minority of studies used ordinal sub-group categories of frailty, the rationale of where to dichotomise is outlined in the results section.

### Outcomes

We evaluated pre-specified outcomes of interest: (i) Mortality (in-hospital, 28-day, 90-day, and 1-year), (ii) Disability Scores: modified Rankin Score (mRS), National Institute of Health Stroke Scale (NIHSS) score, (iii) Complications: stroke recurrence, symptomatic intracranial haemorrhage, post-stroke delirium, myocardial infarction.

Other relevant outcomes described, including activities of daily living (ADLs), quality of life, including mental well-being, were incorporated into the discussion.

### Literature sources

The relevant articles were systematically identified from four databases: PubMed, EMBASE (Ovid), Scopus and Web of Science. Searches used the broad terms: (‘stroke’ OR ‘cerebrovascular accident’) AND (‘frailty’) AND (‘thrombectomy’ OR ‘thrombolysis’). Specific search strategies were tailored for each database inquiry, developed using Cochrane search filters. Subsequently published papers were included when the search period elapsed through forward searching. Manual searching of references from included studies and relevant review articles was also performed.

### Study selection and data collection

We used Rayyan software for de-duplication, and title and abstract screening [20]. Full-text screening and subsequent data extraction was completed using a standardised data collection form with data extracted by one study investigator, and checked by a second researcher.

Data extracted included author name, publication year, country, study design, frailty assessment tool and definition of frailty, sample sizes, age, primary outcomes (as referenced above) and additional outcomes described in the individual studies.

The Risk of Bias in Observational Studies of Exposures (ROBINS-E) tool was used to assess the risk of bias of the studies included in the analysis [21]. Furthermore, we conducted a leave-one-out sensitivity analysis to evaluate for the influence of individual studies on the pooled results.

### Synthesis of results data

Results were synthesised and presented with tabulated results on quantitative measures of morbidity and mortality outcomes, prior to meta-analysis. We categorised frailty as a binary variable based on definitions used in included studies. In cases where frailty was classed as mild, moderate, or severe, those participants within the ‘severe frailty’ group were classified as frail.

### Statistical analysis

Data was extracted from the identified studies where numbers of relevant events were described in the frail and non-frail populations. Given that considerable study heterogeneity was anticipated, random-effects models were utilised for meta-analysis. The restricted maximum likelihood estimator was used to calculate the between-study variance [22]. I^2^ statistics were also calculated to determine the variability in effect estimate due to between-study heterogeneity. Meta-analyses were performed where there were two or more studies using the same population inclusion criteria. Pooled outcomes from the included studies were reported as risk ratios.

Funnel plot asymmetry was assessed visually and using Egger’s test, where 10 or more studies were included in the meta-analysis to identify any small study effects. Two-tailed tests were used, and a p-value of 0.05 was taken as the limit of statistical significance. Statistical analyses were performed using the metafor package [23], using R Statistical Software (v4.4.3; R Foundation for Statistical Computing, 2025).

## Results

**Figure 1:**
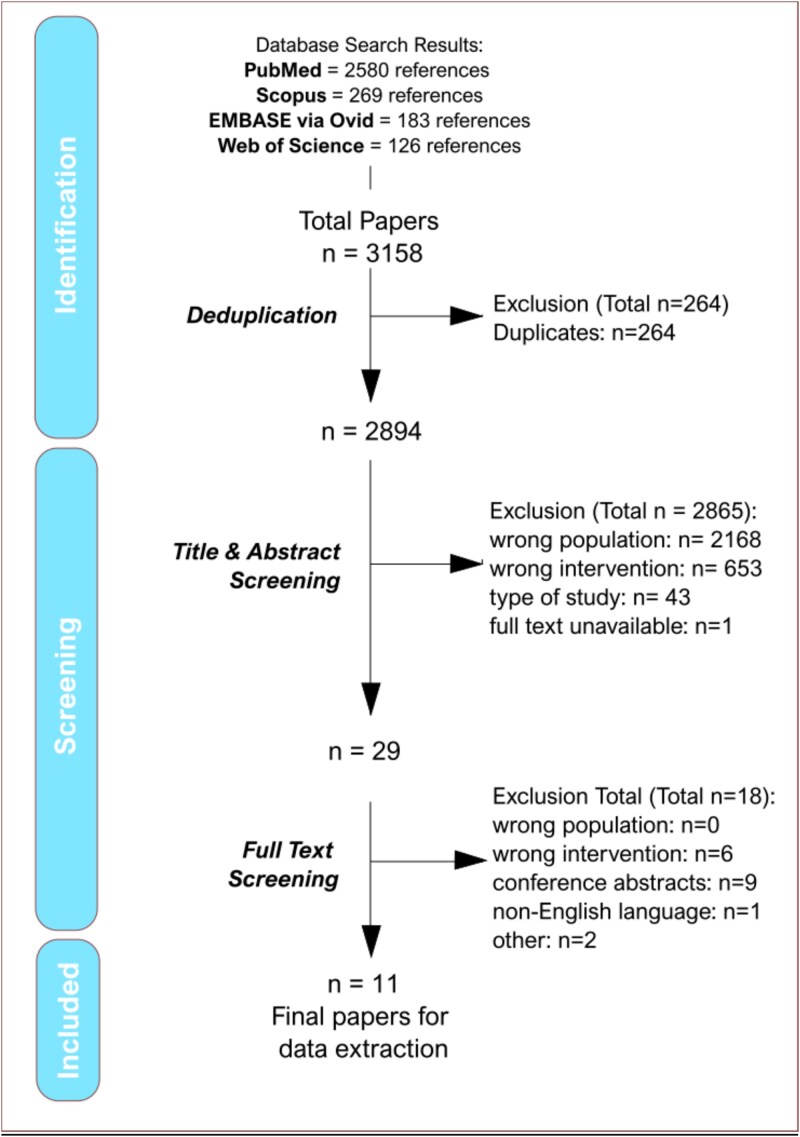
Flow chart of literature search and study selection.

Following deduplication of the database identified records, screening was conducted on 2894 records by title and abstract. This subsequently identified 29 studies for full-text review. Ultimately, a total of 11 studies [5, 9, 24–32] met the inclusion criteria for the systematic review, though two papers included the same cohort as part of a single centre and multicentre study respectively—when the same outcome was reported in each study, only the results from the multicentre study was used in order to avoid duplication of results [27, 28]. Consequently, 194,699 participants were included. In addition, nine conference abstracts were identified as eligible for inclusion but were further excluded due to no recognised full-text publications respectively. The most frequent criteria for exclusion remained a focus on non-acute ischaemic stroke populations (incorrect population) and lack of frailty data (incorrect intervention) (Figure 1).

### Included study characteristics

The characteristics of included studies are summarised in Table 1. Seven studies were single-centre hospital-based studies, and four were multicentre studies. The sample sizes varied from 21 to 133,190 participants, with a median sample size of 198 (inter-quartile range 1489). The prevalence of frailty was 37.2% [frail (n = 72,311), non-frail (n = 122,096)].

**Table 1:** Characteristics of included studies

Study	Country	Study Design	Setting	Follow-Up Period	Sample Size	Age (years)	Female Sex (%)		Frailty Scoring Tool	Frailty Definition	Thrombolysis		Thrombectomy		Primary Outcomes	Variables adjusted for
							Frail	Non-Frail			Frail	Non-Frail	Frail	Non-Frail		
Evans, 2020	England	Retrospective Cohort Study	Single centre	2013–2016	63	≥75	NR	NR	CFS	‘Frail’: 5–8 ‘Non-Frail’: 1–4 Excluded 9	27	36	NA	NA	NIHSS	Cardiovascular risk factors (Diabetes mellitus, Hypertension, Atrial Fibrillation), door-to-needle time, baseline stroke severity (baseline NIHSS), age, sex.
Joyce, 2022	Northern Ireland	Retrospective Cohort Study	Single centre	2014–2018	175	72	49	60	Cumulative Deficit Frailty Index - 33 items	‘Frail’: ≥0.24 ‘Non-Frail’: <0.24	19	55	49	126	mRS at 90 days, 24-hour NIHSS. mortality 90 days, sICH, successful recanalisation.	Age, Sex, baseline NIHSS, thrombolysis.
Pilotto, 2022	Germany	Retrospective Cohort Study	Single Centre	2015–2018	102	>65 years old	46.9	40	MPI	‘Frail’: ≥0.34 ‘Non-Frail’: <0.33	32	70	9	22	3 & 12 month all-cause mortality, mRS, complications.	Age, sex, baseline NIHSS, vascular comorbidities, time-to-treatment.
Schnieder, 2021	Germany	Retrospective Cohort Study	Single centre	2015–2019	318	≥65	69.95	57.6	HFRS	‘Frail’: ≥5 ‘Non-Frail’: <5	50	137	80	238	mRS at discharge and 3 months, mortality at 3 months, modified treatment in cerebral infarction score (mTICI), pneumonia.	Age, sex, mTICI, ASPECTS, change in NIHSS, time from onset to recanalisation, hemicraniectomy, thrombolysis, hours on ventilation, rates of pneumonia, mRS at discharge, intracranial haemorrhage, Elixhauser and Charlson Comorbidity Index.
Tan, 2022	Singapore	Retrospective Cohort Study	Single centre	2017–2020	198	≥70 years old	61.5	41.5	CFS	‘Frail’: >3 ‘Non-Frail’: 1–3	60	59	104	94	mRS at 90 days, duration of hospitalisation, In hospital mortality, sICH, haemorrhagic transformation, successful reperfusion, carer requirement.	Age, NIHSS, time to intervention.
Tiainen, 2022	Finland	Retrospective Cohort Study	Single centre	2016–2019	159	≥80 years old	88.2	52	CFS	‘Frail’: ≥5 ‘Non-Frail’: 1–4	11	52	34	125	mRS at 90 days, recanalisation, sICH, 1 year survival, return home 12 months post-stroke.	Age, sex, early ischaemic signs in >1/3 of the middle cerebral artery region, thrombolysis, NIHSS prior to treatment, mTICI post-thrombectomy, sICH.
Yang, 2022	China	Retrospective Cohort Study	Single centre	2019–2020	21	65–99	NR	NR	FRAIL Score	‘Frail: 3–5 ‘Non Frail’: 0–2	4	13	0	4	Functional recovery status	-
Gajjar, 2025	United States of America	Retrospective Study	Multi-centre	2017–2021	57,260	≥18	43.39	43.72	mFI-5	‘Frail’: ≥2 ‘Non Frail’: <2	NA	NA	54,195	3065	Mortality, complications, length of stay, discharge disposition and cost of care	Age, different frailty indices
Bahar, 2025	United States of America	Retrospective Study	Multi-centre	2016–2021	133,190	≥18	60.7	50.3	Johns Hopkins Adjusted Clinical Groups (ACG)	‘Frail’: ≥ 1 diagnostic cluster ‘Non Frail’: <1 diagnostic cluster	NA	NA	17,245	115,945	In hospital all-cause mortality Morbidity: periprocedural stroke, seizures, ICH, major adverse cardiac events (MACE), cardiogenic shock, cardiac arrest, MI, PE, DVT, Sepsis HAP, AKI, Length of stay, total hospitalisation charges and cost	Patient level variables, medical comorbidities, socioeconomic status, hospital level variables
Huang, 2025	China	Post hoc analysis – of prospective double blind RCT	Multi-centre	2019–2023	771	≥55	33.6	35.7	mFI-5	‘Frail’: ≥2 ‘Non Frail’: <2	NA	NA	172	599	Functional independence 90 days (mRS) NIHSS score from baseline, 24 hours or 5–7 days post recanalization 90 day mortality Complications: ICH	Patient age, baseline NIHSS, ASPECTS, OTR, eTICI, ASITN/SIR
Schnieder, 2025	Germany	Retrospective Cohort Study	Multi-centre registry	2016–2019	2468	≥65	60.6	53.6	HFRS	‘Frail’: ≥5 ‘Non-Frail’: <5	226	1049	449	2009	NIHSS at discharge, mRS at discharge and 90 days, mortality at 90 days, modified treatment in cerebral infarction score (mTICI).	Age, sex, mTICI, ASPECTS, admission NIHSS, baseline mRS, thrombolysis, hours on ventilation, rates of pneumonia, mRS at discharge, intracranial haemorrhage, Elixhauser and Charlson Comorbidity Index.

Three studies used CFS, while the others adopted HFRS (2), Modified 5-item Frailty Index (mFI-5; 2), Cumulative Deficit Frailty Index (1), Frailty Index and Cumulative Deficit Frailty Index (FRAIL) score (1), Multidimensional Prognostic Index (MPI; 1) and the Johns Hopkins Adjusted Clinical Groups (ACG) system (1) to measure frailty status.

Eight of the studies dichotomised frailty in the primary analysis, with the authors using the following thresholds to differentiate frailty from non-frailty: CFS >3 [24], CFS ≥4 [9, 26], frailty index >0.24 [25], MPI ≥0.34 [32], FRAIL ≥3 [5], Johns Hopkins ACG ≥1 [31] and mFI-5 ≥2 [30]. Three studies reported ordinal categories: both papers by Schnieder et al. used three groups according to HFRS (<5, 5–15 and > 15), but we dichotomised frailty as above ≥5 (corresponding to the combined moderate and high-risk of frailty groups) as this threshold was used to dichotomise frailty in their smaller single-centre cohort study [27, 28]. Gajjar et al. used a four category approach using the mFI-5 (non-frail = 0, pre-frail = 1, frail = 2, severely frail = 3+), and we dichotomised at ≥2 given the very small numbers of non-frail individuals and to be consistent with the majority of other studies where pre-frailty was categorised as non-frail [29].

### Post-reperfusion mortality

**Figure 2:**
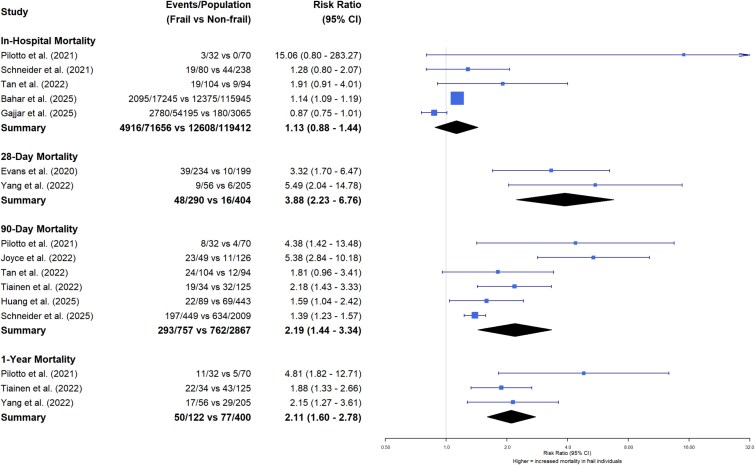
Forest plot of mortality at different time points according to frailty status.

Pre-stroke frailty is associated with increased mortality at three time points: 28 days [2 studies, RR 3.88 (2.23–6.76)], 90 days [6 studies, RR 2.19 (1.44–3.34)] and one year after stroke [3 studies, RR 2.11 (1.60–2.78)] (Figure 2). However, there was no association between pre-stroke frailty and in-hospital mortality [5 studies, RR 1.13 (0.88–1.44).

### Post-reperfusion morbidity

**Figure 3:**
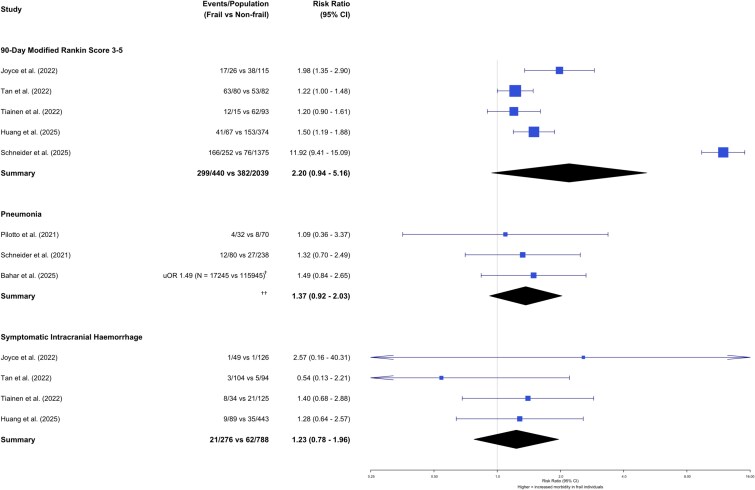
Forest plot of functional disability and post-reperfusion therapy complications according to pre-morbid frailty status (†Indicates no raw data available, ††Indicates no cumulative data calculated due to unavailable raw data).

There was a trend towards premorbid frailty being associated with an increased risk of mRS 3–5 versus mRS 0–2 at 90-days post-reperfusion therapy, though it did not reach statistical significance [5 studies, RR 2.20 (0.94–5.16)]. Similarly, there was no difference in the rates of complications, namely secondary symptomatic intracranial haemorrhage [4 studies, RR 1.23 (0.78–1.96)] and pneumonia [3 studies, OR 1.37 (0.92–2.03)] (Figure 3).

### Risk of bias assessment

The risk of bias assessment highlighted a typically ‘low’ risk of bias across domains 2–7 (bias due to selection of participants, bias in classification of interventions, bias due to deviations from intended interventions, bias due to missing data, bias in measurement of outcomes and bias in the selection of the reported result). However, there was a ‘high’ risk of bias given the baseline demographics were different in each study, with the frail group typically being older. The direction of effect was consistent across the included studies (see Appendix 1 in the Supplementary Data section for the full risk of bias assessment).

Based on this, sensitivity analysis was performed using ‘leave-one-out’ methodology. This found no effect on the statistical significance of the effect sizes for each of the mortality and morbidity criteria listed above.

## Discussion

The outcome of this systematic review and meta-analysis suggests several important findings for individuals with concurrent AIS and frailty undergoing hyperacute reperfusion therapies: Firstly, around one in three individuals receiving reperfusion therapies in AIS had frailty, broadly comparable to the proportion seen in the wider stroke population [6]. Secondly, there was no significant increase in post-procedural complications (post-procedure symptomatic intracerebral haemorrhage and pneumonia) for individuals with frailty compared to those without frailty. Thirdly, there was an increased risk of death in the medium to long-term: 28-day mortality was nearly four-fold higher for individuals with frailty, whilst 90-day and one-year mortality was approximately doubled. Finally, for those who survived, there was no significant difference in the proportion of individuals with a dependent level of disability (mRS 3–5 versus mRS 0–2).

Such findings may have implications for clinical decision-making and case selection for reperfusion therapies in the coming decades. In the UK, the ageing population is anticipated to contribute to increased numbers of strokes, as well as a higher burden of individuals presenting with concurrent frailty [14]. Understanding the balance of benefit and risks in this population will be critical, either in terms of minimising exposure to risks in individuals with a low probability of benefit, or conversely combatting treatment nihilism by ensuring that older individuals who may benefit from reperfusion therapies receive them. Fundamentally, being able to tailor discussion of risks and benefit for the individual with frailty may facilitate more accurate shared decision-making.

There are some areas where future studies could add more detail to understand these relationships. The finding that frailty was associated with increased mortality at 3- and 12-months post-procedure is consistent with disease trajectories in frailty from studies across a range of medical and surgical conditions. Hence, further characterisation of the cause of death—in particular whether cerebrovascular versus non-cerebrovascular in aetiology—may help elucidate the mechanisms contributing to the death and whether the disease trajectory is a direct consequence of the stroke. However, a fundamental appreciation of limited life expectancy post-procedure, regardless of the cause of death, may be sufficient to inform discussions between clinicians and patients/relatives about preferences regarding care and future planning.

Another important consideration is whether a moderate disability (such as a modified Rankin score of three) is necessarily an undesirable outcome. Although many stroke studies have used modified Rankin scores of 0–1 or 0–2 to indicate ‘good’ outcomes, Individuals with higher modified Rankin scores may well be able to return to their usual place of residence, albeit with additional care needs. Whether this represents an acceptable outcome to stroke survivors—as well as whether the costs economics of such an approach—requires further investigation [17].

In addition to the potential clinical implications of our results, these findings also highlight the need for a better understanding of the mechanisms that may underlie the attenuated response to reperfusion therapies in individuals with frailty. The presence of cardiovascular risk factors, which often accompany frailty, has been shown to promote neuroinflammation even in the absence of an acute neurovascular event [33]. Similarly, systemic inflammation with atherosclerosis is not only related to frailty, but also with an increased severity of cerebral small vessel disease, a feature of the ‘frail brain.’ [34–36] These findings suggest how frailty may ‘prime’ the brain for injury and contribute to attenuated outcomes. Indeed, frailty has been found to be associated with reduced salvageable penumbra in hyperacute ischaemic stroke, after adjustment for age, vascular risk factors, leukoaraiosis burden and collateral status [37]. The presence of a potential inflammatory-mediated mechanism underlying these relationships raises the possibility of therapeutic exploited to reduce the impact of frailty on clinical outcomes.

From a practical perspective, this systematic review also highlights the variation in frailty assessment tools used, as well as the time at which frailty was assessed. Given the time-critical nature of reperfusion therapies, any frailty assessment will need to be quick to administer. Other considerations—such as the information typically available in the acute setting, as well as inter-rater reliability of the assessments—will be important to the operationalisation of frailty assessment in hyperacute settings [18, 38]. Furthermore, the findings of the systematic review support the inclusion of frailty—and not solely chronological age—as covariables in future stroke studies.

Although there is a clear trend in the summary results of the meta-analysis, it is based on only a few observation studies and hence the findings should be viewed as proof of principle. Heterogeneity in the frailty assessments used, as well as the outcome measures, also has implications for being able to compare studies. In particular, the different thresholds used to define frailty in the included studies may give rise to some variation: not only may there be effects from such thresholds not being directly comparable (for example, a frailty index threshold of ≥0.24 used in Joyce et al. may not be directly comparable to a FRAIL score threshold of ≥3 used by Yang et al. [5, 25]), but also from variation in thresholds using the same measure (such as the CFS threshold of ≥4 and ≥5 in Tan et al. and Evans et al. respectively [9, 24]). Despite this, most thresholds were based on validated thresholds for the respective scoring tools and typically occurred around a point corresponding to moderate frailty, thereby ensuring a comparable exposure across studies. However, a dichotomised measure is arguably reductive and loses valuable information when treating those with a moderate degree of frailty, and consequently the use of a continuous measure may provide more nuanced information to guide the management of borderline cases that typically have the highest degree of uncertainty relating to clinical decision-making. Validation in a larger prospective study, comparing frailty measures with consistent and comparable thresholds and standardised study endpoints, would be advantageous.

In conclusion, the ageing population and rise in frailty amongst hyperacute stroke presentations represents both a looming challenge for stroke care, as well as an opportunity to develop more tailored hyperacute reperfusion strategies. The findings of this systematic review highlight the additional mortality risk for individuals with frailty, but with no significant difference in functional outcomes or rates of complications amongst those who survive. Although these findings begin to inform clinical decision-making in reperfusion therapy decision-making, they also highlight the need for further studies in this important area.

## Supplementary Material

aa-25-2103-File002_afag080

## References

[1] Clegg A, Young J, Iliffe S et al. Frailty in elderly people. Lancet. 2013;381:752–62. 10.1016/S0140-6736(12)62167-9. 23395245 PMC4098658

[2] Fogg C, Fraser SDS, Roderick P et al. The dynamics of frailty development and progression in older adults in primary care in England (2006–2017): a retrospective cohort profile. BMC Geriatr 2022;22:30. 10.1186/s12877-021-02684-y. PMC8740419 34991479

[3] Court T, Capkova N, Pająk A et al. Frailty index is an independent predictor of all-cause and cardiovascular mortality in Eastern Europe: a multicentre cohort study. J Epidemiol Community Health 2024:jech-2023-221761;79:56–63. 10.1136/jech-2023-221761. 39181708 PMC11671974

[4] Wallis SJ, Wall J, Biram RW et al. Association of the clinical frailty scale with hospital outcomes. Qjm. 2015;108:943–9. 10.1093/qjmed/hcv066. 25778109

[5] Yang F, Li N, Yang L et al. Association of pre-stroke frailty with prognosis of elderly patients with acute cerebral infarction: a cohort study. Front Neurol 2022;13:855532. 10.3389/fneur.2022.855532. 35711265 PMC9196308

[6] Burton JK, Stewart J, Blair M et al. Prevalence and implications of frailty in acute stroke: systematic review & meta-analysis. Age Ageing 2022;51:afac064. 10.1093/ageing/afac064. PMC9037368 35352795

[7] Evans NR, Pinho J, Beishon L et al. Frailty and stroke: global implications for assessment, research, and clinical care-a WSO scientific statement. Int J Stroke 2025;20:905–17. 10.1177/17474930251345295. 40390672 PMC12446689

[8] Kahlon S, Pederson J, Majumdar SR et al. Association between frailty and 30-day outcomes after discharge from hospital. Cmaj. 2015;187:799–804. 10.1503/cmaj.150100. 26009583 PMC4527901

[9] Evans NR, Wall J., To B et al. Clinical frailty independently predicts early mortality after ischaemic stroke. Age Ageing 2020;49:588–91. 10.1093/ageing/afaa004. 31951248

[10] Lewis B, Dohle E, Warburton EA et al. Frailty and early mortality following intracerebral hemorrhage. Cerebrovasc Dis 2024;3:1–12. 10.1159/000541701 39362192

[11] Goyal M, Menon BK, van Zwam WH et al. Endovascular thrombectomy after large-vessel ischaemic stroke: a meta-analysis of individual patient data from five randomised trials. Lancet. 2016;387:1723–31. 10.1016/S0140-6736(16)00163-X. 26898852

[12] Hacke W, Kaste M, Bluhmki E et al. Thrombolysis with alteplase 3 to 4.5 hours after acute ischemic stroke. N Engl J Med 2008;359:1317–29. 10.1056/NEJMoa0804656. 18815396

[13] Taylor-Rowan M, Cuthbertson G, Keir R et al. The prevalence of frailty among acute stroke patients, and evaluation of method of assessment. Clin Rehabil 2019;33:1688–96. 10.1177/0269215519841417. 30971115

[14] King D, Wittenberg R, Patel A et al. The future incidence, prevalence and costs of stroke in the UK. Age Ageing 2020;49:277–82. 10.1093/ageing/afz163. 31957781 PMC7047821

[15] Sobolewski P, Brola W, Wilczyński J et al. Cerebral thrombolysis in rural residents aged ≥ 80. Clin Interv Aging 2020;Volume 15:1737–51. 10.2147/CIA.S256070. PMC7522422 33061326

[16] Hill G, Regan S, Francis R et al. Research priorities to improve stroke outcomes. Lancet Neurol 2022;21:312–3. 10.1016/S1474-4422(22)00044-8. 35305334 PMC8926410

[17] Evans NR, Minhas JS, Beishon LC et al. Stroke medicine is frailty medicine: clinical and research priorities for frailty in stroke. Cerebrovasc Dis 2025;15:1–12. 10.1159/000545288. 40090312

[18] Evans NR, Fearon P, Beishon L et al. The importance of frailty in stroke and how to measure it. Stroke. 2024;56:e8-e11. 10.1161/STROKEAHA.124.048424. 39234696

[19] Page MJ, McKenzie JE, Bossuyt PM et al. The PRISMA 2020 statement: an updated guideline for reporting systematic reviews. Bmj. 2021;29:n71. 10.1136/bmj.n71. PMC8005924 33782057

[20] Ouzzani M, Hammady H, Fedorowicz Z et al. Rayyan-a web and mobile app for systematic reviews. Syst Rev 2016;5:210. 10.1186/s13643-016-0384-4. 27919275 PMC5139140

[21] Higgins JPT, Morgan RL, Rooney AA et al. A tool to assess risk of bias in non-randomized follow-up studies of exposure effects (ROBINS-E). Environ Int 2024;186:108602. 10.1016/j.envint.2024.108602. 38555664 PMC11098530

[22] Viechtbauer W . Bias and efficiency of meta-analytic variance estimators in the random-effects model. Journal of Educational and Behavioral Statistics 2005;30:261–93. 10.3102/10769986030003261.

[23] Viechtbauer W . Conducting meta-analyses in R with the metafor package. J Stat Softw 2010;36:1–48. 10.18637/jss.v036.i03.

[24] Tan BYQ, Ho JSY, Leow AS et al. Effect of frailty on outcomes of endovascular treatment for acute ischaemic stroke in older patients. Age Ageing 2022;51:afac096. 10.1093/ageing/afac096. 35486669

[25] Joyce N, Atkinson T, Mc Guire K et al. Frailty and stroke thrombectomy outcomes-an observational cohort study. Age Ageing 2022;51:afab260. 10.1093/ageing/afab260. 35150584

[26] Tiainen M, Martinez-Majander N, Virtanen P et al. Clinical frailty and outcome after mechanical thrombectomy for stroke in patients aged ≥ 80 years. J Stroke Cerebrovasc Dis 2022;31:106816. 10.1016/j.jstrokecerebrovasdis.2022.106816. 36215902

[27] Schnieder M, Bähr M, Kirsch M et al. Analysis of frailty in geriatric patients as a prognostic factor in endovascular treated patients with large vessel occlusion strokes. J Clin Med 2021;10:2171. 10.3390/jcm10102171. 34069797 PMC8157268

[28] Schnieder M, Metz H, Baehr M et al. Frailty's influence on older stroke patients: neurological outcome and mortality after endovascular treatment in stroke: a national German stroke registry analysis. Eur Stroke J 2025;10:1312–9. 10.1177/23969873251344202. 40497328 PMC12158978

[29] Gajjar AA, Chen JY, Prabhala T et al. National assessment of frailty impact on endovascular mechanical thrombectomy for minor to moderately severe large vessel occlusions per NIHSS. Interv Neuroradiol 2025;2:15910199251342854. 10.1177/15910199251342854. PMC12129947 40452410

[30] Huang L, Gong C, Qiu Z et al. Association between premorbid frailty status and functional independence in acute ischemic stroke patients following endovascular treatment in the late window. Clin Interv Aging 2025;20:523–35. 10.2147/CIA.S504456. 40330269 PMC12053410

[31] Bahar AR, Bahar Y, Kidess G et al. Association of frailty with outcomes in patients with large vessel occlusion stroke undergoing mechanical thrombectomy. Neuroradiology. 2025;67:845–54. 10.1007/s00234-025-03562-9. 39998617

[32] Pilotto A, Brass C, Fassbender K et al. Premorbid frailty predicts short- and long-term outcomes of reperfusion treatment in acute stroke. J Neurol 2022;269:3338–42. 10.1007/s00415-022-10966-7. 35039903

[33] Drake C, Boutin H, Jones MS et al. Brain inflammation is induced by co-morbidities and risk factors for stroke. Brain Behav Immun 2011;25:1113–22. 10.1016/j.bbi.2011.02.008. 21356305 PMC3145158

[34] Evans NR, Tarkin JM, Walsh J et al. Carotid Atheroinflammation is associated with cerebral small vessel disease severity. Frontiers in Neurology [Original Research] 2021;12:2021. 10.3389/fneur.2021.690935. PMC8438317 34531813

[35] Appleton JP, Woodhouse LJ, Adami A et al. Imaging markers of small vessel disease and brain frailty, and outcomes in acute stroke. Neurology. 2020;94:e439–52. 10.1212/WNL.0000000000008881. 31882527 PMC7080284

[36] Evans NR, Bhakta S, Zeicu C et al. Carotid atherosclerosis shows distinct patterns of Atheroinflammation and microcalcification relating to frailty and multimorbidity. Cerebrovasc Dis 2025;55:176–83. 10.1159/000546563. 40472822

[37] Dohle E, Lewis B, Agarwal S et al. Frailty reduces penumbral volumes and attenuates treatment response in hyperacute ischemic stroke. Age Ageing 2024;53:afae266. 10.1093/ageing/afae266. PMC11645531 39656764

[38] Hanlon P, Welsh SA, Evans NR. Constructing a quality frailty index: you get out what you put in. Age Ageing 2024;53:afad248. 10.1093/ageing/afad248. 38266125

