# Supplementary material for: Treatment-modifying effects of frailty on stroke reperfusion therapy outcomes: a systematic review and meta-analysis
Source: Age Ageing. 2026 Apr 10;55(4):afag080. doi: 10.1093/ageing/afag080 (PMC13070556; doi:10.1093/ageing/afag080)
Supplement: aa-25-2103-File002_afag080 [file aa-25-2103-file002_afag080.docx]

**Treatment-modifying Effects of Frailty on Stroke Reperfusion Therapy Outcomes: A Systematic Review and Meta-analysis: Supplementary material**

| Study | Prospective (P) / retrospective (R) | Domain 1 | Domain 2 | Domain 3 | Domain 4 | Domain 5 | Domain 6 | Domain 7 | Overall | Direction of effect |
| --- | --- | --- | --- | --- | --- | --- | --- | --- | --- | --- |
| Bahar (2025) | R | High | Low | Low | Low | Some concerns | Low | Low | High | Unknown |
| Evans (2020) | P | High | Low | Low | Low | Low | Low | Low | High | Frailty worse |
| Gajjar (2025) | R | High | Low | Low | Low | Some concerns | Low | Low | High | Unknown |
| Huang (2025) | R | High | Low | Low | Low | Low | Low | Low | High | Frailty worse |
| Joyce (2022) | R | High | Low | Low | Low | Low | Low | Low | High | Frailty worse |
| Miranda (2024) | R | High | Low | Low | Low | Low | Low | Some concerns | High | Unknown |
| Pilotto (2022) | R | High | Low | Low | Low | Low | Low | Low | High | Frailty worse |
| Schneider (2021) | R | High | Low | Low | Low | Low | Low | Low | High | Frailty worse |
| Schneider (2025) | R | High | Low | Low | Low | Low | Low | Low | High | Frailty worse |
| Tan (2022) | R | High | Low | Low | Low | Low | Low | Low | High | Frailty worse |
| Tiainen (2022) | R | High | Low | Low | Low | Low | Low | Low | High | Frailty worse |
| Yang (2022) | P | High | Low | Low | Low | Low | Low | Low | High | Frailty worse |

Appendix 1: Risk of bias assessment. Domain 1: Bias due to confounding; Domain 2: Bias due to selection of participants; Domain 3: Bias in classification of interventions; Domain 4: Bias due to deviations from intended interventions; Domain 5: Bias due to missing data; Domain 6: Bias in measurement of outcomes; Domain 7: Bias in selection of the reported result.
